# Enhanced Electrochemical Sensing of Oxalic Acid Based on VS_2_ Nanoflower-Decorated Glassy Carbon Electrode Prepared by Hydrothermal Method

**DOI:** 10.3390/bios14080387

**Published:** 2024-08-09

**Authors:** Mengfan Wu, Zhuang Sun, Peizheng Shi, Ningbin Zhao, Kaiqiang Sun, Chen Ye, He Li, Nan Jiang, Li Fu, Yunlong Zhou, Cheng-Te Lin

**Affiliations:** 1 Joint Centre of Translational Medicine, The First Affiliated Hospital of Wenzhou Medical University, Wenzhou 325000, China; wumengfan@nimte.ac.cn; 2 Zhejiang Engineering Research Center for Tissue Repair Materials, Joint Centre of Translational Medicine of Wenzhou Institute, University of Chinese Academy of Sciences, Wenzhou 325000, China; 3 School of Ophthalmology and Optometry, School of Biomedical Engineering, Wenzhou Medical University, Wenzhou 325035, China; 4 Qianwan Institute, Ningbo Institute of Materials Technology and Engineering (NIMTE), Chinese Academy of Sciences, Ningbo 315201, China; sunzhuang@nimte.ac.cn (Z.S.); shipeizheng@nimte.ac.cn (P.S.); zhaoningbin@nimte.ac.cn (N.Z.); sunkaiqiang@nimte.ac.cn (K.S.); yechen@nimte.ac.cn (C.Y.); lihe@nimte.ac.cn (H.L.); jiangnan@nimte.ac.cn (N.J.); 5 Center of Materials Science and Optoelectronics Engineering, University of Chinese Academy of Sciences, Beijing 100049, China; 6 Key Laboratory of Marine Materials and Related Technologies, Zhejiang Key Laboratory of Marine Materials and Protective Technologies, Ningbo Institute of Materials Technology and Engineering (NIMTE), Chinese Academy of Sciences, Ningbo 315201, China; 7 College of Materials and Environmental Engineering, Hangzhou Dianzi University, Hangzhou 310018, China

**Keywords:** oxalic acid, vanadium disulfide, electrochemical sensor, differential pulse voltammetry

## Abstract

Oxalic acid (OA) is a predominant constituent in kidney stones, contributing to 70–80% of all cases. Rapid detection of OA is vital for the early diagnosis and treatment of kidney stone conditions. This work introduces a novel electrochemical sensing approach for OA, leveraging vanadium disulfide (VS_2_) nanoflowers synthesized via hydrothermal synthesis. These VS_2_ nanoflowers, known for their excellent electrocatalytic properties and large surface area, are used to modify glassy carbon electrodes for enhanced OA sensing. The proposed OA sensor exhibits high sensitivity and selectivity across a wide linear detection range of 0.2–20 μM, with an impressively low detection limit of 0.188 μM. The practicality of this sensor was validated through interference studies, offering a promising tool for the early diagnosis and monitoring of kidney stone diseases.

## 1. Introduction

Kidney stones are a common urological disorder resulting from the abnormal accumulation of crystalline substances in the kidneys [1]. Various factors contribute to the formation of kidney stones, including metabolic disorders (such as hyperparathyroidism, hypercortisolism, and hyperglycemia), prolonged bed rest, nutritional deficiencies (vitamin B6 deficiency, magnesium-deficient diet), urinary tract obstruction, infection, foreign bodies, and drug use [2]. Calcium oxalate is the most common component of kidney stones, accounting for 70–80%, while other components include uric acid, calcium phosphate, and cystine (an amino acid) [3]. In China, the prevalence of kidney stones in adults is approximately 5–10% [4]. Therefore, developing methods and devices for detecting kidney stone components is of great significance for the diagnosis and prevention of kidney stone diseases.

Currently, the diagnosis of kidney stones mainly relies on medical imaging techniques such as X-ray, ultrasound, and CT scans, which can identify the shape and location of stones within the body [5,6,7]. For stone composition analysis, ex vivo stone component testing is the primary method. The above-mentioned methods are effective diagnostic tools only after the kidney stones have formed into larger shapes. In the early stages of kidney stone formation, when symptoms are not obvious, the detection of kidney stone components in the body can be inferred by observing the crystalline morphology in concentrated urine under an optical microscope [8]. Routine urine tests do not include the detection of kidney stone indicators. At present, 24 h urine biochemical tests are used in clinical practice to detect some kidney-stone-related indicators [9,10]. However, these tests still require liquid chromatography, mass spectrometry, and other methods and equipment, which have issues such as complex sample processing and detection methods, large equipment size, difficulty in achieving multi-channel testing, and long testing time for individual samples. Currently, it is challenging to implement these tests on a large scale in hospital laboratories and even more difficult to meet the growing demand for point-of-care testing (POCT) [11,12,13,14,15]. Oxalic acid (OA) is the main component of the most common type of calcium oxalate kidney stones [16]. As an electrochemically active substance, OA has an oxidation potential of around 1.3 V, making it suitable for quantitative detection using electrochemical voltammetric methods [17].

Transition metal dichalcogenides (TMDCs) have attracted significant attention due to their unique morphology and graphene-like properties [18]. These materials exhibit excellent chemical, physical, optical, mechanical, magnetic, and electrical characteristics [19,20]. Among TMDCs, vanadium disulfide (VS_2_) stands out for its remarkable properties, including excellent conductivity, a high aspect ratio, ultrathin edges, and favorable mechanical characteristics [21,22]. Recent advancements in first-principles theoretical calculations and experimental research have demonstrated that two-dimensional layered VS_2_ fulfills the essential criteria for an effective electrochemical sensor [23,24].

In this study, we utilize a hydrothermal method to synthesize VS_2_ nanoflowers and employ them to modify GCEs for the sensitive detection of OA. Various characterization techniques, including scanning electron microscopy (SEM), transmission electron microscopy (TEM), X-ray diffraction (XRD), Raman spectroscopy, and X-ray photoelectron spectroscopy (XPS), were used to investigate the morphology, structure, and composition of the synthesized VS_2_ nanoflowers. The electrochemical performance of the VS_2_ nanoflower-modified GCE was evaluated by differential pulse voltammetry (DPV). The results demonstrate that the VS_2_ nanoflower-modified GCE exhibits excellent sensitivity and selectivity towards OA detection, with a wide linear range and low detection limit. Furthermore, the practical applicability of the sensor was validated through interference studies. This work provides a promising approach for the development of electrochemical sensors based on VS_2_ nanoflowers for sensitive OA detection, which has potential applications in the early diagnosis and monitoring of kidney stone diseases.

## 2. Materials and Methods

### 2.1. Materials

Sodium metavanadate (Na_3_VO_4_·12H_2_O), thioacetamide (TAA), and OA were purchased from Sigma-Aldrich (Shanghai, China). Phosphate buffered saline (PBS) was obtained from Sangon Biotech (Shanghai, China). Artificial urine samples (pH 5.7) were purchased from Phygene (Fuzhou, China). Screen-printed electrodes were acquired from Poten (Shanghai, China). All other chemicals were of analytical grade and used without further purification. Deionized water was used throughout the experiments.

### 2.2. Synthesis of VS_2_ Nanoflowers

VS_2_ nanoflowers were synthesized using a hydrothermal method. First, 0.9 g of sodium metavanadate and 0.9 g of thioacetamide were dissolved in 30 mL of deionized water and stirred magnetically at room temperature for 1 h (stirring speed: 630 rpm). After stirring, the solution was transferred to a 50 mL Teflon-lined stainless steel autoclave and heated at 160 °C for 24 h. The product was collected from the autoclave, washed with deionized water, and centrifuged at 5000 rpm to collect the precipitate. The precipitate was then vacuum-dried at 50 °C for 12 h to obtain VS_2_ nanoflowers [12,25].

### 2.3. Preparation of VS_2_ = −Modified GCE (VS_2_/GCE)

A 1 mg/mL dispersion of VS_2_ nanoflowers in ethanol was prepared for electrode modification. GCEs (3 mm diameter) were polished with 0.05 μm alumina slurry, rinsed with deionized water, and cleaned. The cleaned GCEs were then activated by repetitive cyclic voltammetry (CV) scanning in 0.5 M sulfuric acid from −1 to 1 V at a scan rate of 100 mV/s. A 6 μL aliquot of the VS_2_ ethanol dispersion was drop-cast onto the GCE and vacuum-dried at 45 °C for 5–10 min to form the modified electrode.

### 2.4. Electrochemical Measurements

All electrochemical measurements were performed using a three-electrode system with a Shanghai Chenhua CHI660E electrochemical workstation. The VS_2_/GCE, a saturated calomel electrode (SCE), and a platinum plate were used as the working electrode, reference electrode, and counter electrode, respectively [26,27]. A 1× PBS solution was used as the electrolyte. CV tests were conducted between −0.2 and 0.6 V at a scan rate of 100 mV/s for five cycles. Differential pulse voltammetry (DPV) was performed from 0.8 to 1.5 V with a pulse period of 0.5 s, step potential of 4 mV, and amplitude of 5 mV. In order to prevent the variation in current due to the use of modified electrodes of different sizes, which would make it impossible to compare the performance of sensors, the current work uniformly uses electrochemical current density for normalized representation, with an electrode area of 0.0707 cm^2^ (a circle with a diameter of 3 mm, geometric area of GCE). Electrochemical impedance spectroscopy (EIS) was carried out with a 10 mV AC voltage over a frequency range of 0.1 to 100 kHz.

### 2.5. Characterization

The morphology and structure of the synthesized VS_2_ nanoflowers were characterized using field emission scanning electron microscopy (FE-SEM, QUANTA 250 FEG, FEI, Hillsboro, OR, USA), high-resolution transmission electron microscopy (HRTEM, JEM-2100F, JEOL, Tokyo, Japan), energy-dispersive X-ray spectroscopy (EDX), and selected area electron diffraction (SAED). X-ray diffraction (XRD, D8 Advance, Bruker, Berlin, Germany) patterns were obtained to analyze the crystal structure of VS_2_. Raman spectroscopy (Renishaw in Via Reflex, Renishaw plc, Wotton-under-Edge, London, UK) and X-ray photoelectron spectroscopy (XPS, Axis SUPRA+, Shimadzu, Japan) were employed to investigate the chemical composition and oxidation states of the elements in VS_2_.

### 2.6. Electrochemical Sensing of OA

The electrochemical sensing performance of the VS_2_/GCE towards OA was evaluated using DPV. The effect of various parameters, such as the amount of VS_2_ modifier, pH, and scan rate, on the sensing performance was investigated. To study the effect of the modifier amount, VS_2_/GCEs were prepared with different VS_2_ loadings (1 to 7 μg), and their responses to OA were compared. The influence of pH on the sensing performance was examined by varying the electrolyte pH from 4 to 8.

### 2.7. Interference Study and Real Sample Analysis

To assess the selectivity of the VS_2_/GCE, an interference study was conducted by introducing common interfering substances into the electrolyte containing OA. The anti-interference ability of the sensor was evaluated by comparing the current responses on an aerometric I-t curve in the presence of interfering substances.

## 3. Results and Discussion

### 3.1. Morphology and Structure Characterization of VS_2_ Nanoflowers

**Figure 1:**
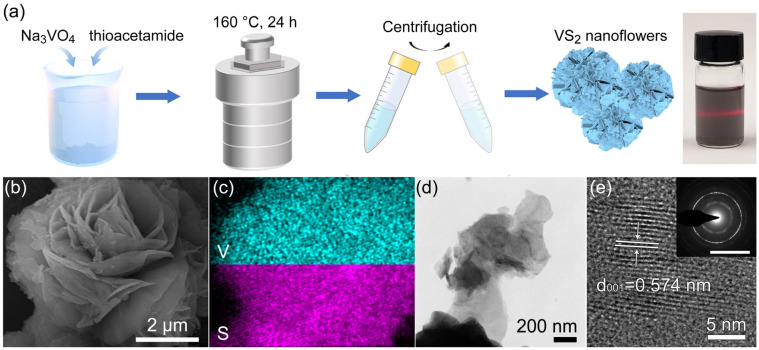
Morphology and structure characterization of hydrothermally synthesized VS_2_ nanoflowers. (**a**) Schematic illustration of the preparation process and digital photograph of the VS_2_ dispersion with a red laser beam passing through. (**b**) SEM image revealing the nanoflower-like morphology of VS_2_. (**c**) Corresponding EDS mapping demonstrating the homogeneous distribution of vanadium and sulfur elements. (**d**) TEM image displaying the edge of the VS_2_ nanoflower. (**e**) HRTEM image exhibiting well-defined lattice fringes, with an interlayer spacing of 0.574 nm. Inset: SAED pattern confirming the polycrystalline nature of VS_2_ nanoflowers.

The preparation process of the hydrothermally synthesized VS_2_ nanoflowers is illustrated in Figure 1a. The digital photograph of the VS_2_ dispersion shows a well-dispersed solution with a red laser beam passing through, indicating the formation of a stable colloidal suspension. The SEM image (Figure 1b) reveals that the synthesized VS_2_ exhibits a unique nanoflower-like morphology, consisting of numerous interconnected nanosheets. The EDX mapping (Figure 1c) demonstrates the homogeneous distribution of vanadium and sulfur elements throughout the nanoflower structure, confirming the successful synthesis of VS_2_.

The TEM image (Figure 1d) provides a bright field image of the edge of VS_2_ nanoflowers, revealing its intricate nanosheet structure. The HRTEM image (Figure 1e) shows well-defined lattice fringes, indicating the high crystallinity of the synthesized VS_2_. It is revealed that the crystalline structure of VS_2_ nanoflowers exhibits an interlayer spacing of approximately 5.74 Å [28]. This value matches the original VS_2_ (001) interplanar spacing (5.76 Å) [29], suggesting that the nanoflowers retain a similar crystallographic arrangement to the bulk material. The selected area electron diffraction (SAED) pattern exhibits concentric rings, suggesting the polycrystalline nature of the VS_2_ nanoflowers. The diffraction rings can be indexed to the (001), (100), and (110) planes of hexagonal VS_2_, which is consistent with the XRD results discussed later.

The unique nanoflower morphology of VS_2_ can be attributed to the hydrothermal synthesis conditions and the presence of thioacetamide (TAA) as the sulfur source. During the hydrothermal process, vanadium ions react with sulfur ions released from the decomposition of TAA, leading to the formation of VS_2_ nanosheets. As the reaction progresses, these nanosheets self-assemble and interconnect, giving rise to the nanoflower structure. This three-dimensional hierarchical architecture provides a large surface area and abundant active sites, which are beneficial for electrochemical sensing applications.

The formation of the VS_2_ nanoflower structure can be further explained by considering the growth mechanism. Initially, vanadium ions (V^4+^) and sulfur ions (S^2−^) combine to form VS_2_ nuclei. These nuclei serve as growth sites for the subsequent formation of VS_2_ nanosheets. As the hydrothermal reaction proceeds, the nanosheets continue to grow and expand in a two-dimensional manner, eventually interconnecting and assembling into the nanoflower morphology. The driving force behind this self-assembly process can be attributed to the minimization of surface energy and the van der Waals interactions between the VS_2_ nanosheets.

### 3.2. Chemical Composition and Crystal Structure Analysis of VS_2_ Nanoflowers

**Figure 2:**
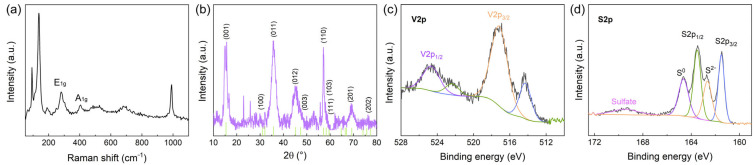
Chemical composition and crystal structure analysis of VS_2_ nanoflowers. (**a**) Raman spectrum and (**b**) XRD of VS_2_. High-resolution (**c**) V2p and (**d**) S2p XPS spectrum of VS_2_.

To further investigate the chemical composition and crystal structure of the synthesized VS_2_ nanoflowers, Raman spectroscopy, XRD, and XPS were employed. Figure 2a presents the Raman spectrum of VS_2_ nanoflowers, which exhibits two prominent peaks at approximately 281 and 405 cm^−1^. These peaks can be assigned to the E_1g_ and A_1g_ vibrational modes of VS_2_, respectively [30]. The E_1g_ mode corresponds to the in-plane vibrations of the V-S bonds, while the A_1g_ mode represents the out-of-plane vibrations of the S atoms [31]. The presence of these characteristic peaks confirms the formation of VS_2_ and is consistent with previous reports on VS_2_ nanostructures.

The XRD pattern of VS_2_ nanoflowers (Figure 2b) displays several sharp diffraction peaks, indicating the high crystallinity of the synthesized material. The peaks at 2θ values of 15.3°, 35.8°, 45.7°, and 56.2° can be indexed to the (001), (100), (012), and (110) planes of hexagonal VS_2_ (JCPDS card No. 89-1640), respectively. The strong intensity of the (001) peak suggests that the VS_2_ nanoflowers have a preferred orientation along the c-axis, which is perpendicular to the basal plane of the VS_2_ layers. The absence of peaks associated with impurities or other vanadium sulfide phases demonstrates the phase purity of the synthesized VS_2_ nanoflowers.

XPS analysis was conducted to investigate the elemental composition and oxidation states of vanadium and sulfur in the VS_2_ nanoflowers. The XPS spectrum of V2p unveiled two prominent peaks centered at 524.8 and 517.4 eV [32,33]. These peaks are assigned to V2p_1/2_ and V2p_3/2_, respectively, affirming the existence of the V^4+^ oxidation state [33]. Also, the XP spectrum exhibits two smaller peaks located at 522.3 and 514.3 eV, suggesting the presence of V^2+^ ions [34]. The emergence of these peaks might be due to the reducing influence of organic amines and the potent reducing effect of hydrogen sulfide, which is produced from the decomposition of thioacetamide during the synthesis process. The XPS spectrum of S2p shows peaks at 161.5 and 163.1 eV, which can be assigned to S2p_3/2_ and S2p_1/2_, respectively [35]. Furthermore, the peak appearing at 162.6 eV can be ascribed to the presence of sulfur ions in a low coordination state at the surface [36]. Meanwhile, the peak at around 169.6 eV can be assigned to the sulfate species. Peaks located at 164.5 eV are attributed to the S^0^ species [37]. These results suggested that the VS_2_ sample has experienced minor oxidation and has become contaminated with elemental sulfur and sulfates as a consequence of being exposed to atmospheric conditions. The XPS results confirm the successful formation of VS_2_ and are in good agreement with the Raman and XRD analyses.

The Raman, XRD, and XPS results collectively provide valuable insights into the chemical composition and crystal structure of the hydrothermally synthesized VS_2_ nanoflowers. The presence of characteristic Raman peaks and the well-defined XRD diffraction pattern confirm the formation of highly crystalline VS_2_ with a hexagonal structure. The XPS analysis verifies the oxidation states of vanadium and sulfur, further corroborating the successful synthesis of VS_2_.

The high crystallinity and phase purity of the VS_2_ nanoflowers can be attributed to the optimized hydrothermal synthesis conditions, including the appropriate ratio of precursors, reaction temperature, and duration. The hydrothermal environment facilitates the nucleation and growth of VS_2_ crystals, promoting the formation of a well-defined layered structure. Moreover, the absence of impurities or other vanadium sulfide phases highlights the effectiveness of the synthesis method in producing high-quality VS_2_ nanoflowers.

The chemical composition and crystal structure of VS_2_ nanoflowers play a crucial role in their electrochemical sensing performance. The highly crystalline nature of VS_2_ ensures efficient electron transfer and enhances the conductivity of the modified electrode. The layered structure of VS_2_ provides a large surface area and abundant active sites for the adsorption and oxidation of OA, leading to improved sensitivity and selectivity. Additionally, the presence of V^4+^ and S^2−^ in their respective oxidation states contributes to the electrochemical activity and stability of the VS_2_ nanoflowers, making them suitable for electrochemical sensing applications.

### 3.3. Electrochemical Behavior of VS_2_/GCE and Optimization of Sensing Conditions

**Figure 3:**
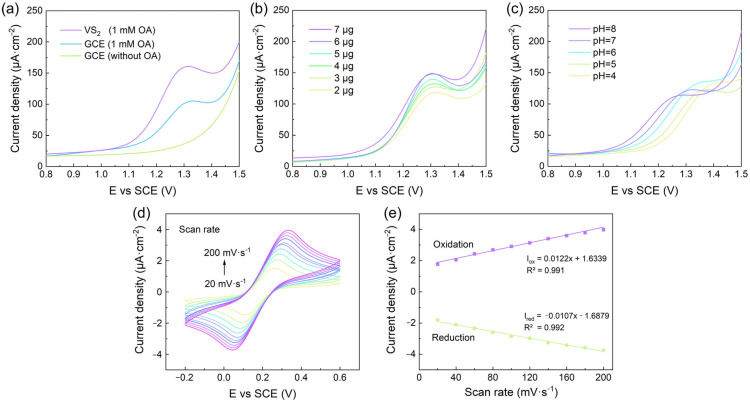
Electrochemical behavior of VS_2_/GCE and optimization of sensing conditions. (**a**) DPV curves of bare GCE and VS_2_/GCE in the presence and absence of 1 mM OA. (**b**) Effect of VS_2_ modifier amount on the oxidation peak current of OA. (**c**) Influence of pH on the oxidation peak current of OA. (**d**) CV curves of VS_2_/GCE in the presence of 10 mM [Fe(CN)_6_]^3-/4-^ at various scan rates (20–200 mV/s). (**e**) Linear relationship between the oxidation and reduction peak currents and the scan rate.

To evaluate the electrochemical performance of the VS_2_/GCE towards OA sensing, DPV measurements were conducted in the presence and absence of OA. As shown in Figure 3a, the bare GCE exhibits a weak oxidation peak for OA, indicating its limited sensitivity (Figure S1). In contrast, the VS_2_/GCE displays a significantly enhanced oxidation peak current density, suggesting the superior electrochemical activity of VS_2_ nanoflowers towards OA oxidation. The improved performance can be attributed to the large surface area, abundant active sites, and excellent electron transfer capability of the VS_2_ nanoflowers, which facilitate the adsorption and oxidation of OA on the electrode surface.

To optimize the sensing performance of the VS_2_/GCE, several key parameters, including the amount of VS_2_ modifier, pH of the electrolyte, and scan rate, were investigated. Figure 3b illustrates the effect of the VS_2_ loading on the oxidation peak current density of OA. As the amount of VS_2_ increases from 2 to 7 μg, the peak current density gradually increases, reaching a maximum value at 6 μg. At this loading level, the electrode surface is thoroughly modified, which maximizes the response current. If the loading is less than 6 μg, the electrode surface may not be fully modified, resulting in insufficient active sites for the electrochemical reaction. This can lead to reduced sensitivity and a less efficient response to the analyte. Conversely, loading more than 6 μg can cause an excessive accumulation of the modified materials on the electrode surface. This accumulation increases the interface resistance, which in turn affects the electron transfer kinetics, potentially leading to peak broadening. Therefore, 6 μg of VS_2_ was chosen as the optimal modifier amount for subsequent experiments.

The influence of pH on the electrochemical response of the VS_2_/GCE towards OA was examined by varying the pH of the supporting electrolyte from 4 to 8. As depicted in Figure 3c, the oxidation peak current of OA reaches its maximum value at pH 6, indicating the optimal pH condition for OA sensing. The observed pH dependence can be attributed to the protonation and deprotonation of OA, which affects its electrochemical behavior [38]. At lower pH values, the protonation of OA hinders its oxidation, resulting in a lower peak current. Conversely, at higher pH values, the deprotonation of OA may lead to the formation of oxalate ions, which have a different oxidation potential and kinetics compared to the neutral form of OA. Consequently, pH 6 was selected as the optimal pH for further experiments.

The electrochemical behavior of VS_2_/GCE was assessed by using 10 mM [Fe(CN)_6_]^3−/4−^ containing 0.1 mM KCl as redox solution at different scan rates ranging from 20 to 200 mV s^−1^ (Figure 3d). The observed peak current (I_ox_ and I_red_) was increased linearly with the square root of scan rates as shown in Figure 3e, indicating that VS_2_/GCE was controlled by diffusion [39]. The electroactive surface area (ESA) was determined using the Randle–Sevcik equation [40,41], which allowed for the calculation of the ESA based on the reduction peak current. The calculated ESA for the VS_2_/GCE was found to be 0.088 cm^2^, which is notably larger than the geometric area of the unmodified GCE (0.071 cm^2^). This increase in ESA highlights the significant enhancement provided by the nanomaterial-modified electrodes, which can be attributed to the high specific surface area and conductivity of the nanomaterials.

The optimization of the sensing conditions, including the VS_2_ modifier amount, pH, and scan rate, plays a crucial role in achieving the best performance of the VS_2_/GCE for OA detection. By carefully tuning these parameters, the sensitivity, selectivity, and reproducibility of the sensor can be significantly enhanced. The improved electrochemical response of the VS_2_/GCE under optimized conditions can be attributed to the synergistic effect of the unique nanoflower morphology, high surface area, and excellent electron transfer properties of VS_2_, along with the favorable pH environment and adsorption-controlled kinetics.

### 3.4. Analytical Performance and Practical Application of the VS_2_/GCE Sensor

**Figure 4:**
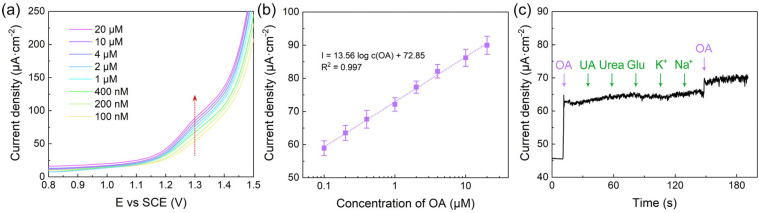
Analytical performance and practical application of the VS_2_/GCE sensor. (**a**) DPV curves of the VS_2_/GCE in the presence of different concentrations of OA (0–20 μM). (**b**) Calibration plot of the oxidation peak current vs. OA concentration. (**c**) Interference study showing the relative response current of the VS_2_/GCE sensor in the presence of various interferents (10 times the concentration of OA).

The analytical performance of the VS_2_/GCE sensor towards OA detection was evaluated using DPV under optimized conditions. Figure 4a presents the DPV curves of the VS_2_/GCE in the presence of different concentrations of OA ranging from 0 to 20 μM. As the concentration of OA increases, the oxidation peak current exhibits a notable increase, indicating the sensitive response of the VS_2_/GCE sensor to OA. The logarithmic linear relationship between the oxidation peak current and OA concentration is depicted in Figure 4b. The calibration plot displays excellent linearity over the concentration range of 0.2 to 20 μM. The limit of detection (LOD) for OA on the VS_2_/GCE sensor is determined to be 188 nM (3 times the standard deviation of the zero concentration). The calibration equation is I = 13.56 log c(OA) + 72.85, with a correlation coefficient R^2^ of 0.997. These results demonstrate the superior analytical performance of the VS_2_/GCE sensor in terms of sensitivity, linear range, and low detection limit.

To assess the selectivity of the VS_2_/GCE sensor, an interference study was conducted by examining the influence of potentially interfering substances on the electrochemical response current of OA. Figure 4c illustrates the relative response current of the VS_2_/GCE sensor in the presence of various interferents, including 2 μM uric acid, urea, glucose, K^+^, and Na^+^, at a concentration 10 times higher than that of OA (0.2 μM). The results reveal that the presence of these interferents has a negligible impact on the current. This observation suggests that the VS_2_/GCE sensor exhibits excellent selectivity towards OA. To verify the reliability of the sensor under actual usage conditions, long-term durability tests and real sample detection using artificial urine samples spiked with OA were conducted. During long-term durability tests, the VS_2_/GCE sensor presented good stability with a recovery ranging from 95% to 105% (Figure S2). In artificial urine, The VS2/GCE sensor exhibited comparable performance to that under laboratory settings, with an LOD of approximately 200 nM (Figure S3). The outcomes suggest that the VS_2_/GCE sensor possesses qualities that render it a viable candidate for practical use. Moreover, with the aim of enhancing applicability in POCT environments, research was directed towards the incorporation of VS_2_ with commercially screen-printed electrodes (SPEs), thereby facilitating ease of use and accessibility. The LOD of VS_2_/SPE sensor remains commendably low at 200 nM. This outcome underscores the remarkable electrochemical activity inherent to the VS_2_ material, even when applied to a different electrode platform (Figure S4).

The analytical performance of the VS_2_/GCE sensor was further compared with other previously reported electrochemical sensors for OA detection, as summarized in Table 1. The VS_2_/GCE sensor exhibits a comparable or even superior performance in terms of linear range, sensitivity, and LOD compared to other state-of-the-art sensors based on various nanomaterials and modified electrodes. This comparison highlights the advantages of employing VS_2_ nanoflowers as an effective electrode modifier for the sensitive and selective detection of OA.

## 4. Conclusions

In summary, a novel approach for the electrochemical detection of OA was presented based on a VS_2_ nanoflower-modified GCE sensor. Synthesized through a hydrothermal method, the VS_2_ nanoflowers exhibit a distinctive nanoflower structure and high crystallinity, providing a substantial surface area and numerous electroactive sites. These features significantly enhance the sensor’s electron transfer efficiency and conductivity. The DPV measurements demonstrated a linear response to OA concentrations ranging from 0.2 to 20 μM, indicating excellent analytical performance. Furthermore, the selectivity study showed that the presence of common interferents had a negligible effect on the sensor’s response to OA, confirming the potential for practical applications in complex sample matrices. The VS_2_/GCE sensor developed in this study offers a promising platform for the detection of OA, paving the way for further advancements in the field of kidney stone diagnostics.

## Figures and Tables

**Table 1:** Comparison of the analytical performance of the VS_2_/GCE sensor with other reported electrochemical sensors for oxalic acid detection.

Modified Electrodes	Measurements	Linear Range	LOD	Ref.
Pd nanocubes/rGO/GCE	DPV	49.5 μM–10 mM	50 μM	[42]
Ag nanorods/graphene/GCE	CV	3–30 mM	40 μM	[43]
CuS nanosphere/GCE	DPV	50–700 μM	35.6 μM	[44]
Graphite/Ag/AgCl	DPV	10–750 μM	3.7 μM	[45]
Ag nanoparticles/N-GO/GCE	Amperometry	10–300 μM	2 μM	[17]
Pt-Pd nanoparticles/chitosan/N-GO/GCE	CV	1.5–500 μM	0.84 μM	[46]
VS_2_/GCE	DPV	0.2–20 μM	0.188 μM	This work

rGO: reduced graphene oxide. N-GO: nitrogen-doped graphene oxide.

## Data Availability

Data are unavailable due to privacy or ethical restrictions.

## Supplementary Materials

The following supporting information can be downloaded at: https://www.mdpi.com/article/10.3390/bios14080387/s1. Figure S1. DPV curves of the bare GCE in the presence of different concentrations of OA; Figure S2. long-term durability of the VS2/GCE sensor; Figure S3. DPV curves of the VS2/GCE in artificial urine with the different concentrations of spiked OA; Figure S4. DPV curves of the VS2/SPE in the presence of different concentrations of OA.
