# Supplementary material for: Enhanced Electrochemical Sensing of Oxalic Acid Based on VS2 Nanoflower-Decorated Glassy Carbon Electrode Prepared by Hydrothermal Method
Source: Biosensors (Basel). 2024 Aug 9;14(8):387. doi: 10.3390/bios14080387 (PMC11352356; doi:10.3390/bios14080387)
Supplement: Supplementary file 1 [file biosensors-14-00387-s001.zip › biosensors-3099263-supplementary.pdf]

biosensors

Supporting information for

Enhanced Electrochemical Sensing of Oxalic Acid Based on VS_2 Nanoflower-Decorated Glassy Carbon Electrode Prepared by Hydrothermal Method

Mengfan Wu [1,2,3], Zhuang Sun [4], Peizheng Shi [4], Ningbin Zhao [4], Kaiqiang Sun [4], Chen Ye [4,5,6], He Li [4,5,6], Nan Jiang [4,5,6], Li Fu [7,*], Yunlong Zhou [1,2,3,*] and Cheng-Te Lin [4,5,6,*]

[1] Joint Centre of Translational Medicine, The First Affiliated Hospital of Wenzhou Medical University, Wenzhou 325000, China; wumengfan@nimte.ac.cn
[2] Zhejiang Engineering Research Center for Tissue Repair Materials, Joint Centre of Translational Medicine of Wenzhou Institute, University of Chinese Academy of Sciences, Wenzhou 325000, China
[3] School of Ophthalmology and Optometry, School of Biomedical Engineering, Wenzhou Medical University, Wenzhou 325035, China
[4] Qianwan Institute, Ningbo Institute of Materials Technology and Engineering (NIMTE), Chinese Academy of Sciences, Ningbo 315201, China; sunzhuang@nimte.ac.cn (Z.S.); shipeizheng@nimte.ac.cn (P.S.); zhaoningbin@nimte.ac.cn (N.Z.); sunkaiqiang@nimte.ac.cn (K.S.); yechen@nimte.ac.cn (C.Y.); lihe@nimte.ac.cn (H.L.); jiangnan@nimte.ac.cn (N.J.)
[5] Center of Materials Science and Optoelectronics Engineering, University of Chinese Academy of Sciences, Beijing 100049, China
[6] Key Laboratory of Marine Materials and Related Technologies, Zhejiang Key Laboratory of Marine Materials and Protective Technologies, Ningbo Institute of Materials Technology and Engineering (NIMTE), Chinese Academy of Sciences, Ningbo 315201, China
[7] College of Materials and Environmental Engineering, Hangzhou Dianzi University, Hangzhou 310018, China
* Correspondence: fuli@hdu.edu.cn (L.F.); zhouyl@ucas.ac.cn (Y.Z.); linzhengde@nimte.ac.cn (C.-T.L.)

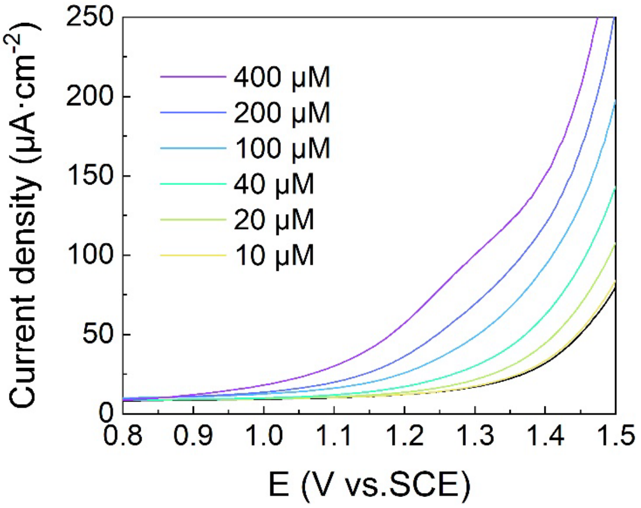

Figure S1. DPV curves of the bare GCE in the presence of different concentrations of OA.

To provide a comprehensive understanding of the performance of the bare GCE for the detection of OA, DPV measurements were also carried out in the presence of different concentrations of oxalic acid (OA). The results indicate that OA concentrations below 100 μM do not produce a discernible peak on the bare GCE. The limit of detection for OA on the bare GCE is approximately 20 μM, which is two orders of magnitude difference from the VS_2/GCE.

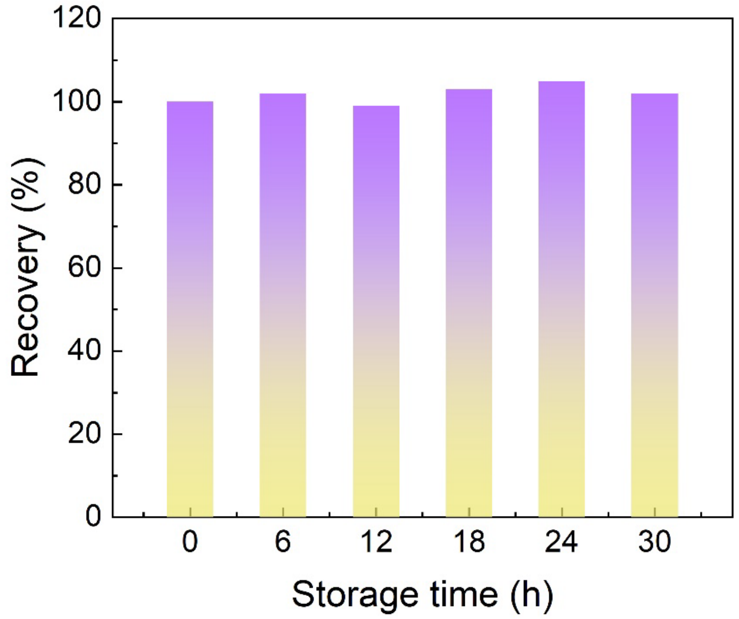

Figure S2. long-term durability of the VS_2/GCE sensor.

The long-term reliability of a sensor are critical parameters that determine its practical utility and effectiveness in real-world applications. To address this, tests were conducted every 6 h to evaluate the performance of the VS_2/GCE sensor within 30 h (OA concentration: 1 μM). The results show that the modified electrode has good stability, with a recovery ranging from 95% to 105%. This indicates that the sensor maintains its sensitivity and reliability over the test period, with minimal degradation in performance.

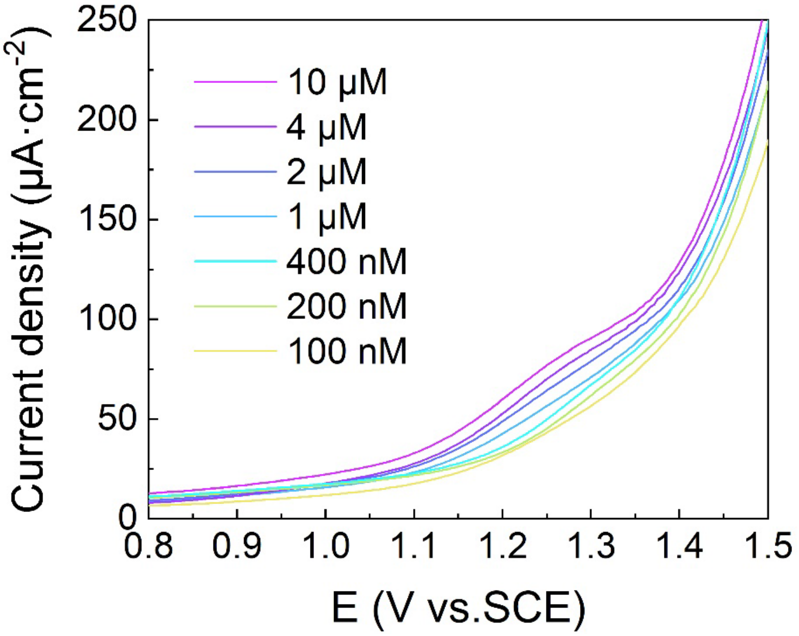

Figure S3. DPV curves of the VS_2/GCE in artificial urine with the different concentrations of spiked OA.

To simulate real-world conditions, experiments were carried out utilizing artificial urine samples spiked with OA. The VS_2/GCE sensor exhibits comparable performance traits in artificial urine as it does under laboratory settings. The sensor's LOD in artificial urine, which is approximately 200 nM, aligns with the sensitivity observed under controlled conditions. This level of consistency across varying environments is indicative of the sensor's robustness and suggests its suitability for dependable deployment in practical applications.

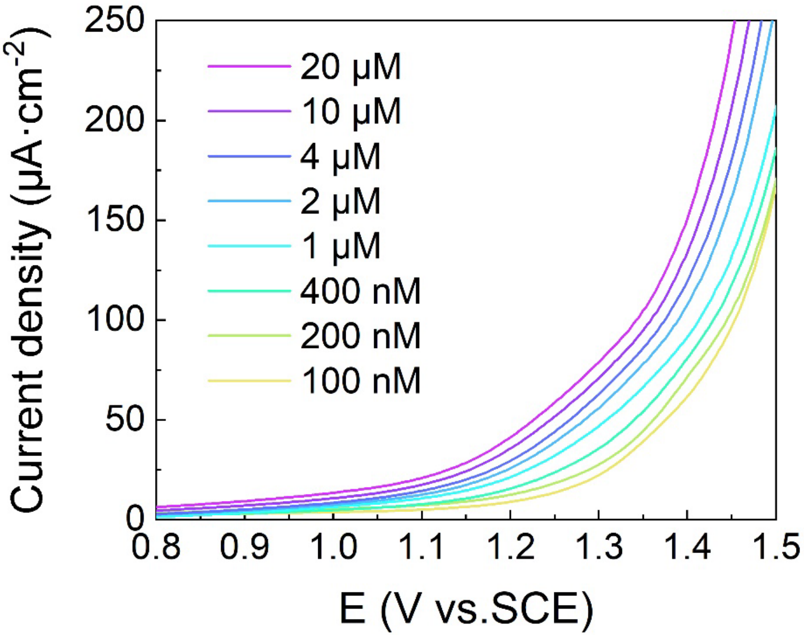

Figure S4. DPV curves of the VS_2/SPE in the presence of different concentrations of OA.

A study was conducted to explore the feasibility of combining VS_2 with commercially available screen-printed electrodes (SPE). This research aimed to assess the potential of integrating VS_2 into existing SPE technology to enhance their performance in various electrochemical applications. All optimization and testing parameters for the VS_2/SPE sensor are aligned with those of the VS_2/GCE sensor to maintain consistency and comparability in this study.

While the conductivity of the SPE is somewhat inferior to that of the GCE, leading to a reduced current response, it is worth to report that the detection limit of the VS_2/SPE sensor remains commendably low at 200 nM. This achievement is a testament to the exceptional electrochemical properties of the VS_2 material, demonstrating its effectiveness even when integrated with a distinct electrode platform. These findings will continue to refine our approach to enhance the sensor's utility in real-world applications, particularly in the context of POCT.
